# Intraoperative Radiotherapy with Balloon-Based Electronic Brachytherapy System—A Systematic Review and First Bulgarian Experience in Breast Cancer Patients

**DOI:** 10.3390/curroncol28050335

**Published:** 2021-10-03

**Authors:** Desislava Kostova-Lefterova, Mariela Vasileva-Slaveva, Svilen Maslyankov, Assia Konsoulova, Margarita Atanasova, Tsvetelina Paycheva, Alexandrina Vlahova, Marusya Genadieva-Yordanova, Ginka Prodanova, Zahari Zahariev, Vasil Pavlov, Georgi Todorov, Boris Vasilev, Kostadin Angelov, Tashko Deliyski, Ivelina Petrova, Desislava Hitova, Ivo Petrov

**Affiliations:** 1 Department of Medical Diagnostic Activities, Medical University—Pleven, 1 Saint Kliment Ohridski Str., 5800 Pleven, Bulgaria; dessi.zvkl@gmail.com; 2 Department of Surgery, University Hospital Acibadem City Clinic Sofia, 66A Tsarigradsko Shose Str., 1784 Sofia, Bulgaria; 3 Women for Oncology—Bulgaria, 9000 Varna, Bulgaria; dr.konsoulova@gmail.com; 4 Department of Surgery, Alexandrovska University Hospital, 1 Georgi Sofiiski Str., 1431 Sofia, Bulgaria; drm@mail.bg (S.M.); prodanovag@gmail.com (G.P.); dr.pavlov11@gmail.com (V.P.); todorovgeo@gmail.com (G.T.); dr.k.angelov@gmail.com (K.A.); 5 Department of Medical Oncology, Oncology Complex Center—Burgas, 86 Demokracia Str., 8000 Burgas, Bulgaria; 6 Department of Intensive Care, Alexandrovska University Hospital, 1 Georgi Sofiiski Str., 1431 Sofia, Bulgaria; meikata@abv.bg; 7 Department of Surgery, Military Medical Academy, 3 Georgi Sofiiski Str., 1431 Sofia, Bulgaria; dr.ts.paycheva@gmail.com; 8 Department of Pathology, Alexandrovska University Hospital, 1 Georgi Sofiiski Str., 1431 Sofia, Bulgaria; alexandrina_vlahova@yahoo.com (A.V.); marusya_zh@abv.bg (M.G.-Y.); 9 Department of Radiotherapy, Uni Hospital, Panagyurishte, 100 Georgi Benkovski Str., 4500 Panagyurishte, Bulgaria; zaharievbg@yahoo.com; 10 Department of Basic Training, University of Telecommunications and Post, 1 Acad. Stephan Mladenov Str., 1700 Sofia, Bulgaria; bovas56@abv.bg; 11 Department of Surgery, Medical University—Pleven, 1 Saint Kliment Ohridski Str., 5800 Pleven, Bulgaria; prof.deliyski@gmail.com (T.D.); ivelina_jurieva@abv.bg (I.P.); 12 Department of Surgical Oncology, Dr. Georgi Stranski University Hospital—Pleven, 8a Georgi Kochev Str., 5809 Pleven, Bulgaria; 13 Department of Radiotherapy, Dr. Georgi Stranski University Hospital—Pleven, 8a Georgi Kochev Str., 5809 Pleven, Bulgaria; drhitova@gmail.com; 14 Department of Medical Physics, Heart and Brain University Hospital, 2 Pierre Curie Str., 5800 Pleven, Bulgaria; evil.petrov@gmail.com

**Keywords:** early breast cancer, intraoperative radiotherapy, electronic brachytherapy, Axxent electronic brachytherapy

## Abstract

(1) Background: We aimed to analyze currently available studies with intraoperative radiotherapy (IORT) as a choice of treatment where the Xoft Axxent^®^ electronic brachytherapy (eBx) system was used as a single-dose irradiation and an exclusive radiotherapy approach at the time of surgery in patients with early breast cancer (EBC). We also compared the results of the systematic review to the Bulgarian experience. (2) Methods and Materials: We performed a systematic review of the studies published before February 2021, which investigate the application of a single-fraction 20 Gy radiation treatment, delivered at the time of lumpectomy in EBC patients with the Xoft Axxent^®^ eBx System. A systematic search in PubMed, Scopus, and ScienceDirect was performed. The results are reported following the PRISMA guidelines. The criteria on patients’ selection for IORT (the additional need for EBRT), cosmetic outcomes, and recurrence rate from the eligible studies are compared to the treatment results in Bulgarian patients. (3) Results: We searched through 1032 results to find 17 eligible studies. There are no published outcomes from randomized trials. When reported, the cosmetic outcomes in most of the studies are defined as excellent. The observed recurrence rate is low (1–5.8%). Still, the number of patients additionally referred to postoperative external breast radiotherapy (EBRT) is up to 31%. Amongst the 20 patients treated in Bulgaria, the cosmetic outcomes are also evaluated as excellent, five of which (25%) are referred for EBRT. Within median follow-up of 39 months, there was one local and one distal recurrence. (4) Conclusions: Current evidence demonstrates the Xoft Axxent^®^ eBx system as a safe and feasible technique for IORT delivery in EBC patients. There are no randomized controlled trials conducted at this time point to prove its long-term effectiveness. Better patient selection and a reimbursement strategy have to be proposed to extend the application of this technique in Bulgaria.

## 1. Introduction

Intraoperative radiotherapy (IORT) is a procedure in which a single-fraction high-dose radiation treatment is delivered directly to the tumor or tumor bed at the time of surgery as a sole local radiation therapy (RT) or selective radiation boost [1,2]. IORT is characterized by an increased therapeutic window [3], limited time for re-population of the remaining tumor cells [4], and possibility for re-irradiation in cases of cancer recurrence [1]. The higher dose applied with this technique (>5 Gy) induces an enhanced immune response in both irradiated and possibly distant unirradiated tumoral volume showing different radiobiological effect [4,5], which is still to be evaluated.

In theory, IORT in early breast cancer (EBC), is the ultimate form of accelerated partial breast irradiation (APBI). IORT provides additional therapeutic benefits by shortening the overall treatment time to one session with a single RT fraction in the operating theater at the time of surgery, sparing further radiation exposure to the normal adjacent tissue. In practice, available data, obtained through randomized trials for some techniques show a link between IORT and higher recurrence rates [6], not allowing wider implementation or introducing the technique as a standard of care in EBC patients as a necessary part of their treatment strategy algorithm. For this reason, extended follow-up analyses and new trials have recently been performed to estimate the exact benefit of each technique. Despite the above-listed IORT showing benefit in nonadvanced primary tumor volume, the issue with recurrence emphasizes the need for unified protocols, careful patient selection, and specific radiotherapy parameters for dose prescriptions, treatment volumes, and dosimetry.

We aimed to analyze currently available studies with the intraoperative radiotherapy (IORT) method of choice where the Xoft Axxent^®^ electronic brachytherapy (eBx) system was used as a single-dose irradiation and an exclusive radiotherapy approach at the time of surgery in patients with early breast cancer (EBC). The results from the proper selection of patients for IORT (no need for EBRT), cosmetic outcomes, and recurrence rate from the eligible studies are compared to the treatment results in Bulgarian patients.

The primary endpoints of the study are feasibility, cosmetic outcomes, and recurrence rate.

## 2. Materials and Methods

This is a systematic review of all trials investigating the feasibility, short, and long-term effects of IORT performed with Axxent^®^ eBx (iCAD, Nashua, NH, USA). The results are reported following PRISMA guidelines. Two independent individuals performed a systematic literature search in PubMed, Scopus, and ScienceDirect. The search strategy included the following terms: “breast cancer, IORT, Axxent”, “breast cancer, IORT, electronic”, and “Axxent, breast”.

The exclusion criteria are the following:

(1) Trials published after February 2021;
(2) Trials in which another technique different from Axxent^®^ eBx is used;
(3) Trials in which the system performing the IORT is not reported [7];
(4) For trials reported multiple times, the latest publication with the highest number of recruited patients was included.

Trials available as an abstract reported at a conference were included if they contain sufficient data to meet inclusion criteria.

We performed a systematic search through 1032 results (Table 1) and found 17 eligible studies (Table 2), with a total of 3889 patients. Another two studies with 30 patients each were published in 2019 [8,9], but without any clinical data or information on the treatment outcome and therefore were excluded from this analysis.

The results from the studies included were compared with the outcomes of patients treated at two Bulgarian hospitals. In Alexandrovska University Hospital, Sofia patients were recruited following the GEC-ESTRO low-risk group criteria [10], while in Georgi Stranski University Hospital, Pleven, the ASTRO recommendations were applied for patients’ selection [11].

In both Bulgarian centers, an atlas of 33 previously prepared brachytherapy plans for breast IORT was used. The plans are developed by Pacific Crest Medical Physics, Chico, CA (PCMP) using a conventional brachytherapy treatment planning system (Varian BrachyVision, incorporating parameters describing the miniature X-ray source data) [12]. The complete set of plans is based on the volume of water used to inflate 3 different sizes of balloon applicators. The balloon size and the quantity of inflated volume are selected to approximate the surgically created cavity. When the cavity size falls within the overlap of two different balloon volume ranges, the smaller balloon is selected, and it is inflated at a higher level. Then, the inflated volume is used to predetermine the optimal dwell positions and the necessary times to deliver the prescribed dose to the balloon’s surface. The different sizes and shapes of the balloon applicators result in different radiation dose ratios between the dose delivered to the tissue in contact with the surface of the balloon applicator and the dose to tissue 1 cm away. A prescription dose of 20 Gy to the balloon’s surface was used (+/− 1 Gy (19–21 Gy) for all pre-outlined plans by PCMP. All plans were prepared to restrict the dose variation to less than 5%. The dose exceeding the prescribed 20 Gy was limited to less than 4 cc of the tissue and less than 1/2 cc of the tissue receiving greater than 22 Gy, with this dose always located at the Lumen–Balloon entrance location. The maximum tissue dose was kept under the 24 Gy (120%) level, while the doses at tissue depth 10 mm were 5–6 Gy for the different plans, depending on the size of the applicator and the inflation volume chosen. 

The X-ray source is positioned in the balloon applicator in the treatment cavity. The dose is delivered in a stepped linear manner to provide conformal distribution through the selected balloon applicator directly to the tissue. Xoft Axxent^®^ X-ray source reference dosimetry is based on an air kerma-rate standard at 50 cm from the source standard, which is provided by the National American Dosimetry Standard Laboratory NIST. A well chamber (HDR-1000; Standard Imaging, Middleton, WI, USA) which comes with every Xoft Axxent^®^ system is calibrated by the accredited dosimetry calibration laboratory to this standard. For techniques with the balloon applicators used in breast IORT, the TG-43 data published by Rivard et al. is used [12]. With the provided well chamber, the output of the currently used individual source is measured as part of the procedure before each irradiation session with treatment times for the plans (that are calculated for nominal source strength) corrected accordingly.

## 3. Results

From all seventeen trials included in our systematic analysis, two studies report results from a 5-year follow-up. Ten studies were conducted in the USA, while seven were conducted in Europe.

**Table 2:** Studies evaluating the effectiveness of the Axxent^®^ eBx System, single dose, 20 Gy.

Study	Country	Patient Number	Median Follow Up (Months)	Mean Time of Irradiation	Cosmetic Outcomes	Recurrence, N (%)	DCIS	Mean Balloon to Skin Distance	EBRT, N (%)
Ivanov (2011) [13]	USA	11	12	22	excellent	0(0)	yes	14 mm	0 (0)
Costa (2015) [14]	PT	30	18	9.1	excellent	1 (3.3)	yes		0 (0)
Dickler (2015) [15]	USA	68	60	13	excellent	4 (5.8)	yes	14 mm	0 (0)
Hanna (2015) [16]	USA	78	12	10.5	good/excellent	0 (0)	yes		9 (11.5)
Zammit (2016) [17]	UK	25	>12		excellent	0 (0)			
Chowdhry (2017) [18]	USA	109	29.9		good/excellent	3 (2.73)	yes		1 (0.91)
Hung-Wen Lai (2017) [19]	TW	261	15			2 (0.76)	yes		8 (3.1)
G. Proulx (2017) [20]	USA	94	26.5		excellent	3 (2.8)	yes	>10 mm	
Olsen (2018) [21]	USA	77			excellent in >50%	0 (0)			
Osorio (2018) [22]	ES	242	16		good		no	>10 mm	63 (26)
Lozares (2018) [23]	ES	150		10.3		0 (0)			
Epstein (2021) [24,25]	USA	1169	52			54 (4.6) *	yes		109 (10.9)
Syed (2018) [26,27] **	USA/PT	1174	19.5	10.5		10 (1.0)	yes	>10 mm	
Vasileva (2019) [28]	BGN	12	2 6 *	12.34	excellent	0 (0)	yes	7–11 mm	1 (8.3)
Crown (2019) [29]	USA	243	46	15–45 mm		3 (1.2)	yes	>7 mm	75 (30.9)
Facer (2019) [30]	USA	100	45.6			2 (2)		>1 mm	
Gullen (2021) [31]	ES	215	24.4	11.8		2(1)	yes		56

* At 5 years follow-up, the recurrence rate is 5.2%. ** In 2020, Syed et al. published a short update of over 1200 patients, but most of the data from the table were not updated [27].

The number of patients in each study ranges from 11 to 1174. The median follow-up calculated from all studies, which have reported follow-up time, is 22.75 months (mean 26.75 months). The mean recurrence rate is 1.5%. Different follow-up time has to be taken into account. The prescription dose at the balloon surface is 20 Gy. Irradiation time ranges from 9 to 45 min. All studies follow the recommendation for at least 7 mm distance from the surface of the applicator to the skin. 

Reporting of the cosmetic outcomes is not uniform across the studies. Lozares et al. [23] report a 6.7% rate of acute dermatitis; Syed [26] report a 3.1% rate of an adverse events. In the study of Dickler [15], overall cosmetics are rated as excellent in 88% of patients. Costa [14] reports only mild skin reactions without any grade 3/4 acute toxicity or delayed healing. In the study of Chowdhry [18], 92.1% of patients have been very pleased with the cosmetics. Overall, the rate of acute reactions after IORT is lower than the observed after EBRT [32,33].

For performing IORT with an Axxent^®^ eBx system in two Bulgarian high volume oncological centers, an ethical committee approval was obtained. All patients included have signed an informed consent. By the end of 2019, 20 patients were treated with Axxent^®^ IORT. The main patient characteristics are shown in Table 3 for Alexandrovska University Hospital, Sofia, and Table 4 for Georgi Stranski University Hospital, Pleven. One patient from each hospital has been diagnosed with invasive lobular carcinoma and is referred to EBRT. The one from Alexandrovska University Hospital, Sofia has received intraoperatively only a 10 Gy boost. Another three patients from Georgi Stranski University Hospital, Pleven have been referred to EBRT either due to positive sentinel node, G3 carcinoma, or close margin revealed on the final pathology report. In three patients, an IORT dose of 10 Gy is prescribed as a boost to the tumor bed due to discordance between intraoperative findings and the applied selection criteria for IORT.

The two Bulgarian centers included in this study are following different treatment protocols. A rapidly frozen section examination is performed in all patients in Alexandrovska hospital, Sofia for the intraoperative evaluation (IOE) of resection margins. Pathological report is given within an hour.

For IOE of the sentinel node, patients are marked with 99mTc-MIBI approximately 24 h before the surgery. To minimize the risk of misdiagnosing micrometastases, the protocol of the College of American Pathologists is followed. One patient had a micrometastasis observed at the final pathology report in one of the SLN (sentinel lymph nodes) investigated. At Georgi Stranski University Hospital, Pleven, the sentinel nodes are evaluated after treatment on paraffin-embedded specimens and reported as “positive” in two patients. 

The mean value for irradiation time is 12.34 min at Alexandrovska University Hospital, Sofia and 13.1 min at Georgi Stranski University Hospital, Pleven varying according to the balloon applicator size and the removed neoplastic mass. The three available sizes of balloon applicators have been used for a range of lumpectomy cavity sizes. At the time of surgery, a Balloon Applicator Specification defines which applicator ought to be implanted following the pathology margin assessment. The size of the resected tumor cavity should always be consistent with the inflated volume range and shape of the selected balloon applicator. The breast size is also taken into consideration for this choice to assure a minimum distance from the balloon surface to the skin surface of more than 7 mm. To deliver radiation treatment, defined dwell positions and dwell times are determined on a separate treatment planning computer using an atlas of 33 pre-designed brachytherapy plans for intra-operative breast treatment, prepared by PCMP. For the same dose prescribed, total irradiation time depends on the size of the lumpectomy cavity and corresponds to the size of the inflated balloon applicator. When IORT is applied only as a boost due to discordance to the selection criteria for IORT, the prescribed dose on the applicator surface is only 10 Gy and is delivered at a shorter (half) time period. Since both hospitals have such patients, this had an impact on the median irradiation time. The dose is computed using the Xoft supplied TG-43 data: a prescription dose of 20 Gy to the surface of the balloon is used for all plans. When needed, the dose is recalculated for 10 Gy for the boost irradiation technique. In all plans, the dose to the surface of the balloon is adjusted to the 20 Gy dose line, within +/− 1 Gy (19–21 Gy). 

The X-ray source is typically limited to deliver up to 10 radiation fractions or 170 treatment minutes.

**Figure 1:**
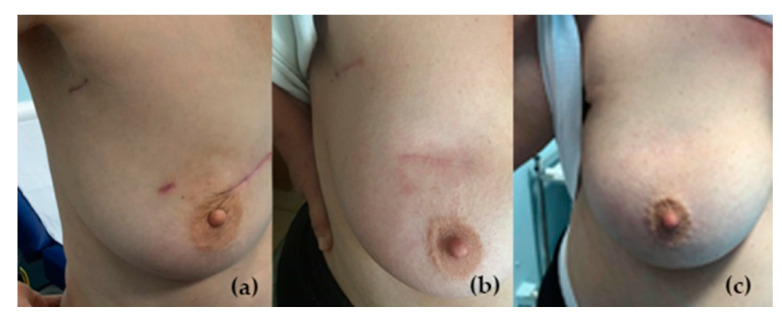
Cosmetic results 6 months (**a**), one year (**b**) and two years (**c**) after treatment.

The median value for the estimated skin dose by the radiotherapy treatment plan is less than 7.5 Gy (6–10 Gy) at Alexandrovska University Hospital, Sofia and less than 6 Gy (3–6.5 Gy) at Georgi Stranski University Hospital, Pleven. No post-treatment radiation-induced skin reactions were observed, and the cosmetic results were evaluated as excellent in both centers (Figure 1). 

Within the median follow-up of 39 months for both hospitals, two events were observed: one in situ recurrence in the same breast in a patient with DCIS and one distant recurrence—skin metastasis.

## 4. Discussion

There are a few techniques for IORT, which can be applied during surgery in patients with EBC: intraoperative radiotherapy with electrons (IOERT) and different brachytherapy techniques—intraoperative high dose rate brachytherapy (IOHDR-brachytherapy) and intraoperative electronic brachytherapy with low energy x-rays (IORT-LEX). These approaches differ according to the ionizing radiation method [34] and could have different effects. The variety of IORT modalities also utilize different approaches to dose prescription and provide a different distribution of the dose in depth of the tumor bed, which further affects the efficacy of the therapy and makes any inter-technique clinical comparison and analysis difficult. 

IOERT is carried out with electron beams produced by linear accelerators generally used for EBRT, or, more recently, with smaller IORT-dedicated accelerators installed in the operation rooms. Modern IOERT machines provide electron beams with energies between 4 and 12 MeV, in steps of 2 MeV or 3 MeV, which results in increased penetration of 7 mm to 1 cm per step [34]. The dose distribution for IOERT is determined by the beam energy and the area covered by the applicator size volume [4]. It delivers the most homogeneous dose distribution compared to interstitial or intracavitary radionuclide brachytherapy and electronic brachytherapy [35].

The IOHDR is defined as the delivery of a single large dose of radiation via photons emitted from a sealed radionuclide brachytherapy source (Iridium-192), usually after maximal tumor resection during surgery [36]. In 2016, the results from a phase I study were reported. IOHDT incorporates a customized CT-based treatment planning and HDR brachytherapy. The multicatheter brachytherapy balloon can be placed in the tumor cavity at the time of surgery or after (in post-pathology cases). The prescribed dose is 12.5 Gy in a single fraction, which is delivered for a median time of 67.2 min. This technique shows promising results and is currently being evaluated in a phase II trial [37]. Another phase III study tests the equivalence of EBRT and APBI using both techniques, external beam and brachytherapy. The APBI does not meet the criteria for equivalence, and the outcomes for brachytherapy techniques are not reported separately [38].

Electronic brachytherapy (eBx) uses miniature LEX sources operating at low kilovoltage energies, <100 kV [39]. EBx machines are also typically mobile and can be operated in a standard treatment room with minimal shielding. There is no risk of radiation leakage in an off state with these devices, no radioactive waste, and no concerns with source transportation [40].

Nowadays, there are already seven eBx devices for IORT available: Intrabeam (Zeiss), Axxent^®^ (Xoft), Papillon+ (Ariane), Photoelectric Therapy (Xstrahl), Esteya (Elekta), SRT 100™ (Sensus Healthcare), and ioRT-50™ unit (Womed cpy). They demonstrate variations in the energy spectra, radial dose function, dose depth, etc. [40]. Four of them—Intrabeam, Axxent^®^ eBx, Papillon+, and ioRT-50™ unit are indicated for IORT in EBC patients. The other three are currently developed and available for treatment of skin cancer. Two of the techniques are developed in the US—the Axxent^®^ eBx system and SRT-100™—and are distributed less in Europe. A detailed comparison of the advantages, disadvantages, and parameters of this system has already been published [40].

The Intrabeam device accelerates a beam of electrons from an electron gun through a thin drift tube down to a gold target, generating low energy X-rays (50 kV, tube current 0.04 mA and 0.1 mmAl HVL). The applicators are for multiple uses, and the life of the source is comparatively long [41]. This is the most studied device with the highest number of treated patients. Its applicability and effectiveness have been investigated in the phase III randomized non-inferiority trial TARGIT-A trial [42]. When first reported, the results of this trial are unconvincing to make the technique a standard of care [43]. The cost-effectiveness analysis has also shown small benefits [44]. In 2019, 5-year follow-up was reached for 95% of patients, and re-analysis was published. The number of local recurrences was 24 (including six ductal carcinomas in situ) of 1140 (2.11%) for TARGIT-IORT versus 11 (including one ductal carcinoma in situ) of 1158 (0.95%) for EBRT. The number of deaths was 42 of 1140 for TARGIT-IORT versus 56 of 1158 for EBRT. All tests have shown non-inferiority of the IORT technique in terms of local recurrence-free survival, invasive local recurrence-free survival, distant disease-free survival, overall survival, and breast cancer mortality [45]. Currently, patients are recruited in prospective registries (ClinicalTrials.gov Identifier: NCT03536897) and trials testing for additional indications of IORT as a boost (ClinicalTrials.gov Identifier: NCT01792726) or in combination with immunotherapy (ClinicalTrials.gov Identifier: NCT02977468).

The first Papillon system with a switchable 30/50 kV X-ray generator and a Chaoul-type (hollow) rod anode tube has been designed in 2009 by the Ariane Company (UK) and approved for treatment of skin and rectal cancer with contact X-ray therapy. Later on, in 2017, a Papillon+ system (with 50 kVp, 0.1–3 mA, 0.7–1.0 mmAl HVL) was introduced for breast IORT [46]. It utilizes reusable serializable spherical applicators in conjunction with the battery-powered mobile X-Ray generator that can produce a dose rate of 8–18 Gy/minute, depending on the distance and added filtration (a dose of 20 Gy in less than 2 min) [47]. Its feasibility is currently investigated in a trial (ClinicalTrials.gov Identifier: NCT03121469).

The ioRT-50™ device was released on the market in 2016 and is recently updated for breast IORT application [46]. The IoRT-50™ device uses a metal-ceramic X-ray tube with Cu target (70 kV, 7 mA, 4.7 mmAl HVL). A set of steam-sterilizable spherical applicators enable accurate adjustment to the target.

The Xoft Axxent^®^ eBx system is a balloon-based technique for IORT that uses a disposable miniature X-ray tube with a tungsten target (50 kV, tube current 0.3 mA and 0.5 mmAl HVL), delivering a higher dose rate [39]. The X-ray source has a near-field dose rate that is at least six times higher beyond the catheter and a slower decreasing depth-dose curve, compared to the Intrabeam System, for example [12].

The Xoft Axxent^®^ eBx system was approved by the Food and Drug Administration (FDA) in the USA for the treatment of EBC in January 2006 [13]. In Europe, it is now in use in 11 countries. Since 2017, it is also available in Bulgaria in four different hospitals across the country. We report the first results of patients that have been treated with this IORT technique in the Department of General Surgery at Alexandrovska University Hospital, Sofia and the Department of Surgical Oncology at Georgi Stranski University Hospital—Pleven. In Bulgaria, the IORT procedure is not reimbursed by the National Health Insurance Fund and, when the applicator is for single-use, the price is paid out-of-the-pocket by the patient.

As a treatment modality that has been assessed and proven effective during long years of investigation and clinical use, EBRT has become the standard of care for all patients with EBC who had undergone breast-conserving surgery [48]. However, EBRT is a procedure that takes 5–6 weeks. Reducing the time of treatment might be a valuable approach in patients with EBC.

The procedure of IORT implies two main features: application during surgery while in the operating theatre and a sole radiotherapy treatment for the patient. Some protocols allow irradiation even 30 days after surgery, but this would change the irradiated volume and result in higher RR [43]. The idea of RT usage during surgery is not new, and it is also applied in other cancer locations [49]. In any case, traditional brachytherapy and IOERT are more widely used than the low-energy X-rays. IOERT has been compared to EBRT in a phase III randomized equivalence trial ELIOT [50]. IOERT was applied with NOVAC 7 (Hythesis, Latina, Italy) or Liac (Info and Tech, Rome, Italy) linear accelerators. The RR reported among patients treated with IOERT was higher than that among patients with EBRT [6]. Long-term results from this trial are published in 2021 with a median follow-up of 12.4 years and confirm the higher RR in the IOERT group, without any difference in OS [51]. 

Traditional breast cancer brachytherapy with radionuclides is a method performed in a way that is technically similar to Axxent^®^ IORT planning and delivery (balloon techniques for breast IORT with X-ray and radioactive sources can be considered identical). Breast cancer brachytherapy with radionuclides is usually fractionated and adjuvant [52]. It differs from Axxent eBx in photon energy spectrum, dose prescriptions, and distribution. This does not allow the extrapolation of the results from the traditional brachytherapy trials to the eBx. [53]. A comparison of the relative biological effectiveness of low energy X-rays of Intrabeam and Axxent^®^ has been published in 2016 and shows that the beam quality of the two sources is very similar [54]. However, dosimetry, planning, and treatment delivery are quite different. Further evaluation and clarified protocols are needed before such results could be transferred and combined the way they are for most of the EBRT machines, for example.

In terms of postoperative EBRT rates, our results are comparable with other studies of Axxent^®^ eBx. One of our patients had an in situ recurrence, while another had a distant skin metastasis. The RR is 10. RR among other trials varies between 0 and 5.8%, with the highest recurrence observed in the study with the most extended follow-up [30]. Similar results are observed also when other techniques are used. In the ELIOT trial, the 5-year IBCR rate is 4.4% [50]. In the final report of the TARGIT A trial at the median follow-up of 8.6 years, the total local recurrence rate was 2.11% for IORT and 0.95% for EBRT [45]. 

Both our patient series and most of the other studies include patients with co-existence of in situ carcinoma as part of the tumor. The latest ASTRO guidelines update on APBI from 2016 allows even low-risk pure DCIS (i.e., screen-detected, low to intermediate nuclear grade, less than or equal to 2.5 cm size, resected with margins negative at ≥3 mm) [11]. 

The indications for IORT applications are still evolving. On one hand, it is hard to reach the RR of EBRT with the partial breast irradiation techniques. On the other hand, current studies are supporting the de-escalation of local treatment in elderly patients, omitting the sentinel node procedure [55] and omitting the postoperative radiotherapy in patients with early stage tumors (pT1, clinically node negative axilla) who are over the age of 70, with no impact on OS or RR [56]. This shows the need for more precise patient selection for each technique. The single-dose IORT has a place in the treatment of low-risk patients, when de-escalation of the radiation treatment volume and dose is possible.

Our patient data series also demonstrate how different protocols, indications, and treatment result in different outcomes of new techniques application. Studies show how IORT—Intrabeam [44] or Xoft eBx [57] can be more cost-effective than EBRT, taking into account RR, complications, and life expectancy. Still, these analyses are performed for specific countries—the United Kingdom and the USA. In Bulgaria, the country with the highest percentage of out-of-pocket expenditure on healthcare—more than 42% for 2017 [58], the results of such analysis would be very different. A study conducted in 2011 and 2012 in Bulgaria showed that 60% of users are paying out-of-pocket payments for both physician services and hospitalization. Of them, about 6% and 10% borrowed money to pay for physician services and for hospitalization and 32% and 6% of the patients forewent physician visits or hospital services, respectively, due to the patient’s inability to pay [59]. This is in addition to the fact that one in every seventh Bulgarian lacks health insurance coverage [60]. Out-of-pocket payments in Bulgaria have become the main source of health care financing [61]. The IORT technique was recently included (2020) as a possible procedure in the clinical pathways for breast cancer treatment, but the price of the pathway has not increased, and this basically means the procedure of IORT is still not reimbursed by the National Health Insurance Fund. There is also no possibility for reimbursement of the price of the balloon applicator. From another point of view, shorter protocols for postoperative RT, such as the FAST-forward protocol [62], are not included as standard protocols for radiotherapy in Bulgaria and are not applied. The standard postoperative RT with more then 20 fractions is free of any charge for the patient and is the best reimbursed method of all RT protocols. The price of the protocols with less than 20 fractions is paid 30% less than the long protocol with more than 20 fractions. Taking all this into consideration, it is not surprising that IORT is applied in a very small number of patients.

Study limitations: There are a few limitations of our study. First of all, there are no published results of any phase III studies using Axxent^®^ eBx for the delivery of IORT in EBC patients, and therefore, all studies included in this review are non-randomized. Most of the studies do not follow single recommendations, which allow the inclusion of younger patients, some patients with pure DCIS, or other deviations from the low-risk group requirements. In addition, the patient number varies a lot across the studies, increasing the bias.

Only 20 patients received treatment for a time period of 32 months in both Bulgarian centers, which is explained by interdisciplinary specialist team availability and financial issues (covered by the patient). A third center in Bulgaria (not being part of this study) has the Intrabeam device [63] for which additional payment for single-use applicators and new X-ray sources is unnecessary. This could be a possible alternative for eligible patients. Still, one therapeutic center cannot address the huge patient load in need of such a treatment.

## 5. Conclusions

Current evidence demonstrates the Xoft Axxent^®^ eBx system as a safe and feasible technique for IORT delivery in EBC patients. There are no randomized controlled trials conducted at this time point to prove its long-term effectiveness. Better patient selection and a reimbursement strategy have to be proposed to extend the application of this technique in Bulgaria.

## Figures and Tables

**Table 1:** Search strategy and key words.

Key Words	PubMed	Scopus	Science Direct	
breast cancer, IORT, Axxent	15	77	49	
breast cancer, IORT, electronic	69	164	288	
breast, Axxent	32	222	116	
total results	116	463	453	1032

**Table 3:** Main characteristics of patients treated with IORT at the Surgery Department, Alexandrovska University Hospital.

Patient Number	Age	Histology	G	ER	PR	HER2	Tumor	Applicator	Applicator	Skin Applicator	Irradiation Dose (Gy)	Irradiation
							Size (mm)	Size (cm)	Volume (cc)	Surface Distance		Time (min)
1	60	IDC *	3	0	0	0	22	4 to 5	50	8	20	13.26
2	58	IDC	2	+	+	1	24	5 to 6	70	7.5	20	18.02
3	73	IDC	3	+	+	0	15	3 to 4	30		20	8.38
4	55	IDC	3	+	+	0	12	3 to 4		8.1	20	8.48
5	59	ILC *	2	+	0	0	14	5 to 6	65	14	10	8.34
6	58	IDC	3	+	+	0	16	3 to 4	40	9	20	13.24
7	68	IDC	2	+	+	0	8	4 to 5	55		20	14.45
8	55	IDC + DCIS	3	+	+	0	16	3 to 4	30	7	20	8.82
9	61	IDC + DCIS	3	0	0	+	20	4 to 5	50	9.3	20	13.74
10	58	IDC	2	+	0	0	12	4 to 5	45	8	20	12.25
11	63	IDC + DCIS	2	+	+	0	11	3 to 4	35	8	20	11.67
12	60	IDC + DCIS	2	+	+	0	13	5 to 6	65	11	20	17.4
Median	59.5						14.5		50	8		12.75
Mean	60.7						15.2		48.6			12.34

* IDC—invasive ductal carcinoma, ILC—invasive lobular carcinoma, DCIS—ductal carcinoma in situ, SLN—sentinel lymph node.

**Table 4:** Main characteristics of patients treated with IORT at the Department of Surgical Oncology of Georgi Stranski University Hospital.

Patient Number	Age	Histology	G	ER	PR	HER2	Tumor Size (mm)	Applicator Size (cm)	Applicator Volume (cc)	Skin Applicator Surface Distance (mm)	Irradiation Dose (Gy)	Irradiation Time (min)
1	56	IDC * DCIS	2	+	+	−	20	4 to 5	60	>10	10	8.28
2	81	ILC LCIS	3	+	0	+	42	4 to 5	60	>10	20	16.56
3	73	IDC DCIS	2,3	+	0	+	20	4 to 5	45	>10	20	13.78
4	50	IDC	1	+	+	0	15	3 to 4	35	>10	20	11.34
5	52	IDC DCIS	2	+	+	0	18	5 to 6	70	>10	10	9.87
6	55	IDC DCIS	2	+	+	+	13	3 to 4	45	>10	20	12.9
7	52	IDC DCIS	2	+	+	+	30	4 to 5	70	>10	20	19.57
8	63	IDC	2	+	+	−	25	3 to 4	40	>10	20	12.28
Median	55.5						20		52.5	>10		12.59
Mean	60.2						22.9		53.1			13.1

* IDC—invasive ductal carcinoma, ILC—invasive lobular carcinoma, DCIS—ductal carcinoma in situ, SLN—sentinel lymph node.

## Data Availability

Eurostat—Tables GaMITg. Out-of-pocket expenditure on healthcare, % share of total current health expenditure: http://appsso.eurostat.ec.europa.eu/nui/show.do?dataset=tepsr_sp310&lang=en, accessed on 24 September 2021.
